# Clinical characteristics of acute hepatitis A outbreak in Taiwan, 2015–2016: observations from a tertiary medical center

**DOI:** 10.1186/s12879-017-2555-x

**Published:** 2017-06-20

**Authors:** Nan-Yu Chen, Zhuo-Hao Liu, Shian-Sen Shie, Tsung-Hsing Chen, Ting-Shu Wu

**Affiliations:** 1 Division of Infectious Diseases, Department of Internal Medicine, Chang Gung Memorial Hospital, Chang Gung University College of Medicine, 5 Fuhsing Street, Kueishan, Taoyuan, 333 Taiwan; 2 Department of Neurosurgery, Chang Gung Memorial Hospital, Chang Gung Medical College and University, 5 FuSing Street, Kueishan, Taoyuan Taiwan; 3 Division of Gastroenterology and Hepatology, Department of Internal Medicine, Chang Gung Memorial Hospital, Chang Gung Medical College and University, 5 FuSing Street, Kueishan, Taoyuan Taiwan

**Keywords:** Acute hepatitis A, HIV infection, Men who have sex with men, Male homosexual, Outbreak

## Abstract

**Background:**

Acute hepatitis A is a fecal-oral transmitted disease related to inadequate sanitary conditions. In addition to its traditional classification, several outbreaks in the men who have sex with men (MSM) population have resulted in acute hepatitis A being recognized as a sexually transmitted disease. However, few studies have clarified the clinical manifestations in these outbreaks involving the MSM population.

**Methods:**

Beginning in June 2015, there was an outbreak of acute hepatitis A involving the MSM population in Northern Taiwan. We conducted a 15-year retrospective study by recruiting 207 patients with the diagnosis of acute hepatitis A that included the pre-outbreak (January 2001 to May 2015) and outbreak (June 2015 to August 2016) periods in a tertiary medical center in Northern Taiwan. Using risk factors, comorbidities, presenting symptoms, laboratory test results and imaging data, we aimed to evaluate the clinical significance of acute hepatitis A in the MSM population, where human immunodeficiency virus (HIV) coinfection is common.

**Results:**

There was a higher prevalence of reported MSM (*p* < 0.001), HIV (*p* < 0.001) and recent syphilis (*p* < 0.05) coinfection with acute hepatitis A during the outbreak period. The outbreak population had more prominent systemic symptoms, was more icteric with a higher total bilirubin level (*p* < 0.05) and had a 7-times higher tendency (*p* < 0.05) to have a hepatitis A relapse.

**Conclusions:**

The clinical course of acute hepatitis A during an outbreak involving the MSM and HIV-positive population is more symptomatic and protracted than in the general population.

**Electronic supplementary material:**

The online version of this article (doi:10.1186/s12879-017-2555-x) contains supplementary material, which is available to authorized users.

## Background

Acute hepatitis A is a fecal-oral transmitted disease related to inadequate hygienic and sanitary conditions. In developed countries, through the elevation of public health standards, hepatitis A has become an imported disease related to travel to endemic countries. Thus, studies have shown decreases in antibody seroprevalence against hepatitis A in developed countries [1], which may also suggest that a large portion of the population is at risk should a hepatitis A outbreak occur. In addition to the traditional transmission route, the role of acute hepatitis A as a sexually transmitted disease (STD) has been recognized due to several reported outbreaks among men who have sex with men (MSM) since the 1990s [2–5]. Previous studies have linked acute hepatitis A to certain risk factors [2, 6–8], including oral-anal sexual practices and intravenous drug use. However, the impact of acute hepatitis A on high-risk populations, particularly the MSM population and those with pre-existing human immunodeficiency virus (HIV) infections, has not yet been clarified.

Acute hepatitis A was endemic in Taiwan before 1995 and has been a notifiable disease according to the Centers of Disease Control (CDC), Taiwan, since 1999 [9]. Since June 1995, when efforts in public health and the free vaccination program for children in 30 indigenous townships were initiated, annual hepatitis A cases have declined from 2.96/100,000 in 1995 to 0.9/100,000 in 2003–2008 [9]. Since then, acute hepatitis A has become a disease mainly related to international travel, and the estimated seroprevalence against hepatitis A has decreased to less than 10% in those under the age of 20 according to the 2011 epidemiologic surveillance [10]. This population is particularly vulnerable to an acute hepatitis A outbreak. According to the CDC, Taiwan, there has been an acute hepatitis A outbreak (serotype IA) in Northern Taiwan since June 2015, notably involving the MSM population, with half of the cases also being infected with HIV [11]. According to the literature, only a few studies have attempted to describe acute hepatitis A in MSM or HIV-infected populations [12, 13]. By the time this manuscript is submitted, more acute hepatitis A cases will have been reported. The aim of this study was to explore the risk differential between the outbreak and sporadic cases in the pre-outbreak period, which may contribute to our understanding of acute hepatitis A involving the MSM and HIV-positive populations.

## Methods

This retrospective study was approved by the Institutional Review Board at Chang Gung Medical Foundation in Taiwan (approval number: 201600805A3). We conducted a 15-year retrospective, single-center study at Chang Gung Memorial Hospital, a tertiary hospital in Northern Taiwan. All patients with a positive or equivocal hepatitis A virus (HAV) immunoglobulin M (IgM) level detected by either an enzyme-linked immunosorbent assay (ELISA) or a radioisotope assay between January 2001 through August 2016 were reviewed for the presence of elevated liver enzymes and symptoms/signs of hepatitis. A case was defined as having acute hepatitis A when there was positive/equivocal HAV IgM together with elevated liver enzymes. Two hundred and sixty-four cases were collected. Twenty-four cases with unavailable/incomplete medical records and 10 cases that came for a health re-check after an acute hepatitis A episode treated elsewhere were excluded. Twenty-three patients with an alternative diagnosis or severe clinically confronting diseases were further excluded (Additional file 1).

A total of 207 acute hepatitis A cases were collected and divided into the following two groups: 163 cases in the pre-outbreak period (January 2001 to May 2015) and 44 cases in the outbreak period (June 2015 to August 2016), as the first case of this national acute hepatitis A outbreak was reported in June 2015 according to a statement by the CDC, Taiwan. Clinical manifestations, laboratory abnormalities, image findings and treatment outcomes were compared between these two groups. The HIV infection status was defined by standard ELISA and Western blotting methods or if the patient was already on combination antiretroviral therapy (cART). Recent travel was defined by domestic or international visits within 2 months before the diagnosis of acute hepatitis A was made. Close contact was defined as living with a confirmed acute hepatitis A case or a person having symptoms or signs of acute hepatitis A in the same household/dormitory or working/studying/in a relationship with a symptomatic person or a confirmed acute hepatitis A case. A recently active syphilis infection was defined as a 4X rise in rapid plasma reagin (RPR)/venereal disease research laboratory (VDRL) test titer or if the patient received benzathine penicillin treatment for syphilis within 6 months of the acute hepatitis A event. In this study, a relapse was arbitrarily defined as a more than 0.5X normal upper limit elevation of either aspartate aminotransferase (AST) or alanine aminotransferase (ALT) during the convalescence phase before liver function normalized.

### Statistical analyses

Analyses were conducted using SigmaPlot 12.0 (Systat Software, Inc., San Jose, California, USA). The differences between groups (before the outbreak and during the outbreak) were tested for significance using the Mann-Whitney Rank Sum test and Pearson’s chi-square test for numerical variables and categorical variables, respectively. Values were reported as the median and ranges, and *p* value of less than 0.05 was considered significant for all statistical tests.

## Results

**Fig. 1:**
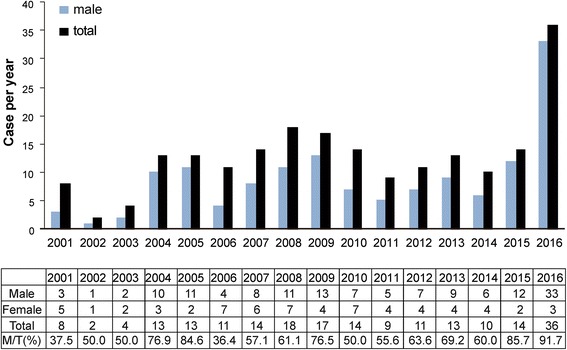
Annual case numbers of acute hepatitis A in the study center from January 2001 to August 2016. M/T: male/total case number ratio. There was an outbreak of acute hepatitis A in the male population in 2015 and 2016

Between June 2015 and August 2016, while Northern Taiwan was experiencing a hepatitis A outbreak, a similar trend was detected at Chang Gung Memorial Hospital (Fig. 1). The number of acute hepatitis A cases increased from 2 to 18 cases/year to 36 cases in 2016 (until Aug 2016). Throughout these 15 years, males often had a stronger association with acute hepatitis A (male/total cases ratio ranging from 36.4 to 84.6%), with a female predominance observed only in 2001 and 2006. The male predominance in acute hepatitis A was further exaggerated during the outbreak (male/total cases ratio of 85.7% in 2015 and 91.7% in 2016), reaching statistical significance (*p* < 0.001, Table 1). As shown in the patient demographic and risk factor analysis (Table 1), recent travel (56/207, 29.4%), particularly international travelling (37/56, 66%), was the most often reported factor among pre-outbreak cases. Conversely, the most frequently reported risk factor for outbreak cases was MSM or HIV infection (40.9%), although recent travel was still reported in a minority of cases (18.2%). Notably, during the outbreak, there were also more cases reported to have close contact to a confirmed acute hepatitis A case or a person having symptoms or signs of acute hepatitis A. The average age of acute hepatitis A cases was quite young (33 and 31 years in the pre-outbreak and outbreak groups, respectively). The prevalence of both HIV (31.8%, *p* < 0.001) and active syphilis infections within 6 months (9.1%, *p* = 0.002) was higher in the outbreak group, suggesting a nature of sexually transmitted hepatitis A during this outbreak. The prevalence of positive hepatitis B surface antigen (HBsAg) was 20.2 and 9.1% in the pre-outbreak and outbreak groups, respectively, and the prevalence of a positive anti-hepatitis C virus (anti-HCV) antibody was 1.2% in the pre-outbreak group and 4.5% in the outbreak group. The difference between the two groups in the underlying viral hepatitis did not reach statistical significance.

**Table 1:** Patient characteristics and demographics

	Total	2001/1–2015/5	2015/6–2016/8	*P* value
Case number	207	163	44	
Gender (M/F^a^)	142/65	102/61	40/4	< 0.001*
Age (median, range)	32 (4–89)	33 (4–89)	31 (10–68)	0.006*
HIV^b^ (n, %)	14 (6.8%)	0	14 (31.8)	< 0.001*
Hospitalization (n, %)	139 (67.1)	110 (67.5)	29 (66.0%)	0.843
Risk factor (n, %)				
Travel	56 (27.1)	48 (29.4)	8 (18.2)	0.136
MSM^c^	14 (6.8)	0	14 (31.8)	< 0.001*
MSM/HIV	18 (8.7)	0	18 (40.9)	< 0.001*
Close contact	8 (3.9)	4 (2.5)	4 (9.1)	0.043*
Foreign workers/indigenous townships	6 (2.9)	4 (2.5)	2 (4.5)	0.463
Positive HBsAg (n, %)	37 (17.9)	33 (20.2)	4 (9.1)	0.087
Positive anti-HCV (n, %)	4 (1.9)	2 (1.2)	2 (4.5)	0.156
Recent syphilis (n, %)	4 (1.9)	0	4 (9.1)	0.002*

Note: ^a^M: male, F: female. ^b^HIV: human immunodeficiency virus, ^c^MSM: men who sex with men, MSM/HIV: MSM or HIV infection

* denotes a significant post hoc comparison between two groups

Considering the clinical manifestations of these cases (Table 2), the duration of the prodromal period was generally about 1 week, with a median of 6 days, and the most common symptoms (>50%) were fever, malaise, nausea/vomiting, poor appetite, abdominal discomfort and jaundice/tea-colored urine. Other symptoms (in order from high to low frequency) included chills (18.4%), arthralgia/myalgia (15.5%), upper respiratory symptoms (14.5%), diarrhea (11.6%), light colored stool (10.1%), headache (8.2%), sore throat (3.9%), hepatic encephalopathy (3.9%), pruritus (2.4%), hiccups (1.5%) and skin rash (0.97%).

**Table 2:** Symptoms and signs of acute hepatitis A

	Total (207)	2001/1–2015/5 (163)	2015/6–2016/8 (44)	*P* value
Prodromal period (median, range)	6 (1–21)	6 (1–21)	6 (2–14)	0.981
Fever (n, %)	116 (56)	86 (52.8)	30 (68.2)	0.050
Chills	38 (18.4)	31 (19)	7 (15.9)	0.668
Malaise	115 (55.6)	85 (52.1)	30 (68.2)	0.042*
Headache	17 (8.2)	15 (9.2)	2 (4.5)	0.532
Nausea/Vomiting	121 (58.5)	93 (57.1)	28 (63.6)	0.361
Poor appetite	108 (52.2)	82 (50.3)	26 (59.1)	0.250
Sore throat	8 (3.9)	6 (3.7)	2 (4.5)	0.675
URI symptoms	30 (14.5)	26 (16)	4 (9.1)	0.272
Arthralgia/myalgia	32 (15.5)	26 (16)	6 (13.6)	0.736
Rash	2 (0.97)	2 (1.2)	0	1.000
Pruritus	5 (2.4)	4 (2.5)	1 (2.3)	0.957
Jaundice or tea-colored urine	139 (67.1)	104 (63.8)	35 (79.5)	0.032*
Jaundice	98 (47.3)	74 (45.4)	24 (54.5)	0.251
Tea-colored urine	104 (50.2)	75 (46)	29 (65.9)	0.014*
Diarrhea	24 (11.6)	16 (9.8)	8 (18.2)	0.114
Abdominal discomfort	106 (51.2)	81 (49.7)	25 (56.8)	0.342
Hiccup	3 (1.5)	3 (1.8)	0	1.000
Light color stool	21 (10.1)	14 (8.6)	7 (15.9)	0.159
Hepatic encephalopathy	8 (3.9)	7 (4.3)	1 (2.3)	1.000

* denotes a significant post hoc comparison between two groups

Table 3 shows the laboratory abnormalities of all cases. The white blood cells were generally within normal limits, with a median of 6.0 X 10^9^/L (range 1.5–21.2 X 10^9^/L). The median hemoglobin (Hb) level was 145 g/L (range 76–186 g/L). Although the platelet count was normal in most cases (median 173 X 10^9^/L, range 57–481 X 10^9^/L), thrombocytopenia was observed in 32.4% of patients (platelet <150 X 10^9^/L). Renal function impairment in acute hepatitis A was more relevant to the patient’s underlying renal disease, with the median serum creatinine level being 74.3 μmol/L (range 26.5–1674.3 μmol/L). Upon comparison of the two study groups, the outbreak group was more likely to have jaundice/tea-colored urine (*p* = 0.032) with a higher total bilirubin level (*p* = 0.009) and a trend of more patients with fever.

**Table 3:** Laboratory and imaging abnormalities of acute hepatitis A

	Total	2001/1–2015/5	2015/6–2016/8	*P* value
WBC count (× 10^9^/L) (median, range)	6.0 (1.5–21.2)	6.1 (1.5–21.2)	5.7 (1.9–13.8)	0.438
Hemoglobin (g/L)	145 (76–186)	141 (76–185)	150 (106–186)	0.079
Platelet count (X 10^9^/L)	173 (57–481)	172 (57–464)	176 (76–481)	0.668
ALT (U/L)	1904 (40–8378)	1849 (40–8378)	2006 (49–5428)	0.595
ALK-P (U/L)	177 (44–933)	171 (44–638)	187 (58–933)	0.216
Total bilirubin (μmol/L)	106.0 (6.9–1145.7)	100.9 (6.8–1145.7)	118.0 (63.3–489.1)	0.009*
Creatinine (μmol/L)	74.3 (26.5–1674.3)	75.1 (26.5–1674.3)	72.9 (38–385.4)	0.240
CT, MRI or Sonography (n, %)	183 (88.4)	145 (89)	38 (86.4)	0.633
Sonography	180 (87)	143 (87.7)	37 (84)	0.525
CT	16 (7.7)	11 (6.7)	5 (11.4)	0.341
Hepatomegaly	32 (15.5)	28 (17.2)	4 (9.1)	0.251
Splenomegaly	56 (27.1)	40 (24.5)	16 (36.4)	0.060
Lymphadenopathy	4 (1.9)	4 (2.5)	0	0.582
Gallbladder wall thickening/ edema	87 (42)	74 (45.4)	13 (29.5)	0.070
Ascites	15 (7.2)	14 (8.6)	1 (2.3)	0.314

Note: *WBC* white blood cells, *ALT* alanine aminotransferase, *ALK-P* alkaline phosphatase, *CT* computed tomography, *MRI* Magnetic resonance imaging

* denotes a significant post hoc comparison between two groups

The most common imaging analysis performed was abdominal sonography (87%) (Table 3), with gallbladder wall thickening/edema being the most common finding (42%). We found that gallbladder wall edema may not be a purely benign sign in acute hepatitis A, as there was one case reported in our study to have gallbladder rupture necessitating surgical intervention. Nevertheless, most cases with gallbladder changes underwent a smooth course, and it did not take long for the edema to resolve (in one case the edema resolved in 3 days). Other common imaging abnormalities were splenomegaly (27.1%), hepatomegaly (15.5%), ascites (7.2%) and lymphadenopathy (1.9%). There were no significant differences in these image abnormalities between the two study groups.

With respect to disease outcome (Table 4), there was an overall complications rate of 6.8% and a mortality rate of 3.9% in the 207 cases. Renal complications were the most common (9/14) and, in some cases, required transient hemodialysis. Two cases exhibited complications with spontaneous bacterial peritonitis, one case had deep vein thrombosis, one case had pulmonary embolism and one case had gallbladder rupture. Mortality and complication rates were not significantly different between the two study groups; however, more hepatitis A relapses during the convalescence phase were observed in the outbreak group (*p* = 0.019). Among these six cases with hepatitis A relapse, two cases were HIV-positive (ages 34 and 36 years), one case had thalassemia (age 33 years), one case had newly diagnosed diabetes mellitus (age 35 years), one case had pre-existing congestive heart failure and chronic renal insufficiency (age 68 years) and one case had underlying hepatitis B virus (HBV)-related liver cirrhosis and hepatocellular carcinoma (age 61 years). Together, these observations suggest a role for the underlying immune status in the course of acute hepatitis A, which was more prominently observed during the outbreak.

**Table 4:** Outcome comparison

	Total (207)	2001/1–2015/5 (163)	2015/6–2016/8 (44)	*P* value
Mortality (n, %)	8 (3.9)	7 (4.3)	1 (2.3)	1.000
Complications	14 (6.8)	13 (8)	1 (2.3)	0.310
Hepatitis A relapse	6 (2.9)	2 (1.2)	4 (9.1)	0.019*

* denotes a significant post hoc comparison between two groups

Considering the cases of mortality (Additional file 1), none of the mortality cases were HIV-positive. Seventy-five percent (6/8) of mortality cases had underlying positive HBsAg, 12.5% (1/8) had underlying positive anti-HCV, 37.5% (3/8) had pre-existing heart diseases and 25% (2/8) had chronic renal diseases. Of note, younger cases of mortality were specifically associated with an underlying HBV infection. In these younger cases, there were issues of multiple substance abuse and poor socioeconomic support, concurrent spontaneous bacterial peritonitis, unstable hemodynamics caused by acute renal failure and upper gastrointestinal bleeding, which deterred the possibility of prompt liver transplantation. There were two young mortality cases with positive cytomegalovirus IgM, although the clinical significance is questionable. In our study, hepatic encephalopathy was an indicator of a grave prognosis, as none of the patients who developed hepatic encephalopathy survived. In contrast, HIV infection did not seem to contribute to mortality in this study.

## Discussion

Acute hepatitis A is an infectious disease whose transmission route, clinical manifestations and risk factors have long been well-characterized. However, the ever-changing population characteristics and the increasing prevalence of people living with HIV have brought clinicians new challenges and insights regarding this disease. The high HIV infection rate during this acute hepatitis A outbreak has drawn our attention, particularly when considering that the prevalence of HIV infection in the general population is as low as 0.1% in Taiwan. Previous studies by Ida et al. showed that HIV-infected patients have a higher HAV viral load, a longer viremia stage and that some HIV-1-infected patients may have HAV viremia even when the clinical symptoms have disappeared and the aminotransferase levels return to normal [14]. Their study may explain why several outbreaks occurred at such frequency among the MSM population or HIV-infected patients, which restates the vulnerability of this population to acute hepatitis A [2–5]. In Taiwan, the low levels of immunity in the young population created the fundamentals for the hepatitis A outbreak, where the seroprevalence of anti-HAV is less than 10% in the general population [10, 15, 16]. We examined acute hepatitis A in 207 patients who received contemporary standard-of-care treatment. The distinct patient demographics before and during the hepatitis A outbreak between June 2015 and August 2016 echo the emerging role of hepatitis A as an STD in the MSM population [17]. The average age of acute hepatitis A cases was quite young in our study, which may reflect the lack of immunity to HAV in young people and recapitulate the infectiousness of HAV in a sensitive otherwise healthy population. The pre-existing male predominance during the pre-outbreak period may reflect the fact that more males are engaged in international business/travel in this society, which was also observed (62%) in a recently published report based on the Taiwan National Health Insurance Research Database [18]. Previous reviews on relapsing hepatitis A have suggested an association between continuing viremia as well as shedding of the virus in stools during the relapse and an interaction between viral infection and immune mechanisms [19]. Our analysis found that acute hepatitis A in the outbreak population had a tendency toward a more icteric and relapsing disease course, which may reflect the frequent existence of immunodeficiency in this population. Previous studies attempting to assess the influence of MSM or HIV infections on acute hepatitis A were mostly limited by the study size. To our knowledge, this is the first study to suggest an impact of MSM or HIV infection on the clinical manifestations of acute hepatitis A.

There are several limitations to this study: 1. This was a single-center study in which the studied population may not represent the entire population of the country. This difference can be observed by the higher positive HBsAg prevalence (17.9%) in the studied population compared to the estimated number of HBV carriers in the entire population (10–13%, between 2.5 and 3 million people in Taiwan [20]) and a higher mortality rate (3.9%) compared to previous reports (1.68% [18], 0.3–0.6% [21]). This is, in part, related to the study center, which was a tertiary referral hospital with patients potentially bearing more co-morbidities. 2. Although we observed a significant increase in the number of MSM and HIV infections during the outbreak, the prevalences of both MSM and HIV infection were underestimated in this study. Before the acute hepatitis A outbreak onset in mid-2015, according to the CDC’s statement on surveillance using phylogenic analysis (information from a CDC public talk held in Taipei on September 10th, 2016), there were only two sporadic HAV/HIV coinfection cases in the past 10 years in the entire population. Therefore, the whole society was not aware of the correlation between HAV/HIV and also HAV/MSM until mid-2015. The percentage of cases that have been tested for their HIV status before the outbreak was only 12.9% (21/163) in our study. During the outbreak, this percentage was increased to 77.3% (34/44), however, there were still cases that were not checked for HIV status either due to a low awareness of HIV/HAV association by the clinicians or a refusal of the test by the patient. Among HIV-positive cases, there were also 21% (3/14) that did not disclose their sexual orientation in our study. Taken together, the data suggested there may be an underestimation of HIV infections in this study, nevertheless, this effect is considered minimal in the pre-outbreak period and in favor of our conclusion in the outbreak period. 3. Because of the nature of retrospective studies and a smaller sample size of HIV-infected patients, we were unable to compare acute hepatitis A head-to-head between HIV-infected and non-HIV-infected patients. Therefore, we are planning to organize a case-controlled study recruiting larger sample sizes in the future in order to better clarify the effects of HIV infection and different cART regiments on the clinical manifestations and outcomes of acute hepatitis A. However, we believe that emphasizing the importance of hepatitis A vaccination among a high-risk population should not be delayed because we have observed a more protracted and symptomatic disease in this population.

The risks of mortality and complications of acute hepatitis A increase with age, pre-existing chronic liver disease and medical co-morbidities [18, 22, 23]. Our study reports that MSM and HIV-positive populations are also at increased risk of developing a more icteric and protracted course. Acute hepatitis A may cause considerable loss to both people and society. In light of outbreak preventions, study by Regan et al. suggested that the critical point of achieving herd immunity to prevent hepatitis A outbreaks is when the immune population to HAV is greater than ~70% [24]. Because viral interactions are not rare [25, 26] and our study described the likely detrimental effects of HIV infection on acute hepatitis A in a sensitive MSM population, these results call for public health awareness and encourage approaches, particularly HAV vaccination, in the MSM/HIV-positive population. This issue may be particularly important in low anti-HAV seroprevalence countries. During the preparation of this manuscript, a new HAV vaccination program targeting HIV-positive population and those recently diagnosed to have other sexually transmitted diseases was launched in October 2016 in Taiwan with an aim to stop this outbreak. Our results offer clinical evidence to recapitulate the importance of preventing a hepatitis A outbreak in the MSM/HIV-positive population.

## Conclusions

In order to achieve control over an acute hepatitis A outbreak, clinical evidence is crucial to persuade high-risk populations into accepting the vaccination policy. MSM and HIV infection are risk factors of acute hepatitis A infection. This study recognized that the clinical course of acute hepatitis A during an outbreak involving the MSM/HIV-positive population is more symptomatic and protracted than the sporadic cases in general population. The result suggests the need to improve the anti-HAV seroprevalence in the MSM/HIV-positive population to reduce the incidence of acute hepatitis A and may be an important reference for developing public health policies.

## Additional file

Additional file 1: Supplementary materials. The clinical information of the excluded cases and causes of mortality in this study are included in the supplementary material. (DOCX 17 kb)

## Abbreviations

ALT: Alanine aminotransferase
AST: Aspartate aminotransferase
cART: Combination antiretroviral therapy
CDC: Centers of Disease Control
ELISA: Enzyme-linked immunosorbent assay
HAV: Hepatitis A virus
Hb: Hemoglobin
HBsAg: Hepatitis B surface antigen
HBV: Hepatitis B virus
HCV: Hepatitis C virus
HIV: Human immunodeficiency virus
IgM: Immunoglobulin M
MSM: Men who have sex with men
RPR/VDRL test: Rapid plasma regain/venereal disease research laboratory test
STD: Sexually transmitted disease
