# Supplementary material for: Clinical characteristics of acute hepatitis A outbreak in Taiwan, 2015–2016: observations from a tertiary medical center
Source: BMC Infect Dis. 2017 Jun 20;17:441. doi: 10.1186/s12879-017-2555-x (PMC5479032; doi:10.1186/s12879-017-2555-x)
Supplement: Supplementary file 1 — Supplementary materials. The clinical information of the excluded cases and causes of mortality in this study are included in the supplementary material. (DOCX 17 kb) [file 12879_2017_2555_MOESM1_ESM.docx]

Supplementary materials

1. Excluded cases:

There were 1 case of PCR-confirmed influenza with myocarditis, one case of CDC -confirmed scrub typhus, one case of CDC-confirmed dengue fever, two cases with underlying primary biliary cirrhosis and fluctuated liver function, one acute hepatitis B case, one chronic hepatitis B with acute flare-up case, three cases with active mycobacterial infection and possible adverse drug effects, one case with culture-confirmed Staphylococcus bacteremia, one case with colon cancer and mechanic bowel obstruction, one cases that only received treatment from another hospital due to acute asthma and possible sepsis with only a mild elevation of liver enzymes, one case with fever of unknown origin for 1 month favored autoimmune disorder with equivocal anti-HAV IgM, one case with initially normal liver enzymes diagnosed to have pneumonia and acute myocardial infarction, one case with abnormal liver function after receiving a Whipple operation, one case with abnormal liver function after receiving cholecystectomy due to common bile duct stones, one case with concurrent systemic lupus erythematosus activity, one case with acute intra-cranial hemorrhage and equivocal anti-HAV IgM, one case with chronic hepatitis C and equivocal anti-HAV IgM, one case with equivocal anti-HAV IgM noted during prolonged hospitalization for congestive heart failure and breast cancer, one case with liver cirrhosis and aortic dissection presenting with abdominal pain and equivocal anti-HAV IgM, and one case with adult Still’s disease.

We did not ignore the possibility of co-existing acute hepatitis A in these excluded cases, however, to avoid the confrontation of the above mentioned conditions which could also cause liver function abnormalities, these cases were not included in our analysis eventually.

Supplementary 2. Underlying diseases and causes of mortality

| Age | Gender | Underlying diseases | Cause of death |
| --- | --- | --- | --- |
| 31 | M | HBV, HCV carrier, drug abuser, alcoholism | Acute renal failure, †UGI bleeding, high amphetamine, morphine level in urine (overdose?) |
| 33 | M | HBV carrier | Liver decompensation, spontaneous bacterial peritonitis, UGI bleeding, ‡CMV IgM(+), CMV Ag(-) |
| 47 | M | HBV carrier, hypertension | Liver decompensation, UGI bleeding, acute renal failure, CMV IgM (+) |
| 54 | M | HBV carrier | Liver decompensation, acute renal failure |
| 67 | F | HBV carrier, diabetes, hypertension, thyroid surgery 30 years ago | UGI bleeding, liver decompensation, acute renal failure |
| 68 | M | Old myocardial infarction with congestive heart failure, pulmonary hypertension, chronic renal failure | Acute on chronic renal failure, heart failure with acute hypoxemic respiratory failure, pneumonia |
| 80 | F | Diabetes, sick sinus syndrome status post pacemaker implantation, chronic renal failure, gout, hypertension | Acute on chronic renal failure, liver decompensation |
| 86 | M | Coronary artery disease, 3-vessel-disease, old cerebrovascular disease | Heart failure, pulmonary embolism with shock |

Note: †UGI bleeding: upper gastrointestinal bleeding. ‡CMV: cytomegalovirus.
